# Deep learning algorithm enables lower limb venous thrombosis detection with CT venography

**DOI:** 10.3389/fcvm.2026.1890645

**Published:** 2026-08-12

**Authors:** Shanshan Shen, Haixiao Yang, Pengchao Wang, Jing Zhang, Jiahao Zhen, Tao Liu

**Affiliations:** 1 Department of Radiology, Hebei General Hospital, Shijiazhuang, Hebei, China; 2 Department of Radiology, The First Hospital of Hebei Medical University, Shijiazhuang, Hebei, China; 3 School of Economics and Management, Hebei University of Science and Technology, Shijiazhuang, Hebei, China

**Keywords:** CT venography, deep learning, DVT, pulmonary embolism, transfer learning

## Abstract

**Introduction:**

Lower limb computed tomography venography (CTV) has low success rates for deep vein thrombosis (DVT) diagnosis. This study applied deep learning to improve DVT identification.

**Methods:**

Our study enrolled 119 positive DVT and 40 negative DVT, 111 positive pulmonary embolism (PE) patients and 20 negative PE. Two algorithms were evaluated: Faster R-CNN trained directly on CTV images, and YOLO11-nano pre-trained on computed tomographic pulmonary angiography (CTPA) then optimized on CTV via transfer learning. Three radiologists (3-5 years' experience) independently interpreted CTV images. Diagnostic performance was compared.

**Results:**

The YOLO11-nano model and Faster R-CNN achieved over 97% accuracy and sensitivity in diagnosing PE. Compared to the three radiologists in diagnosing DVT, YOLO also had an excellent accuracy (92.1%) and sensitivity (93.9%), superior to Faster R-CNN (66% accuracy, 68% sensitivity). The YOLO model exceeded 77% accuracy in pelvic and femoral-popliteal segments and 66.7% in detecting calf segment thrombi.

**Discussion:**

The CTPA-based transfer learning model significantly improved the feasibility of this method in routine CTV diagnostic performance, offering a promising approach for simultaneous PE and DVT detection.

## Introduction

The incidence of deep vein thrombosis (DVT) in the lower extremities is gradually increasing, particularly among patients with post-traumatic fractures, malignancies, or those requiring prolonged bed rest. DVT can be complicated by life-threatening acute pulmonary embolism (PE). Furthermore, the long-term complication of DVT, post-thrombotic syndrome (PTS), significantly impairs patients' quality of life. Therefore, early and accurate detection of DVT is crucial for reducing the risk of complications and improving patient outcomes.

According to Chinese and European expert consensus on the diagnosis of acute DVT (1, 2), primary diagnostic methods include D-dimer testing, Doppler ultrasound, computed tomography venography (CTV), magnetic resonance venography (MRV), and contrast venography. Apart from D-dimer, all others are imaging modalities. However, some patients with acute DVT may present with negative D-dimer results, while others, such as those with tumors, may have persistently elevated D-dimer levels, making it unreliable as a sole indicator. Thus, imaging plays a vital role in DVT diagnosis. For chronic venous obstructive diseases, CTV was considered as the preferred imaging modality.

Lower limb CTV involves one of the longest scanning ranges in whole-body imaging, generating a large number of image slices. In clinical practice, radiologists often prefer using 3D reconstructions for interpretation. However, due to performance limitations of clinical workstations, image reconstruction can be challenging, often requiring excessive time or causing system crashes. Consequently, applying artificial intelligence and deep learning methods for DVT screening could significantly save time for radiologists and clinicians, thereby improving workflow efficiency.

**Figure 1:**
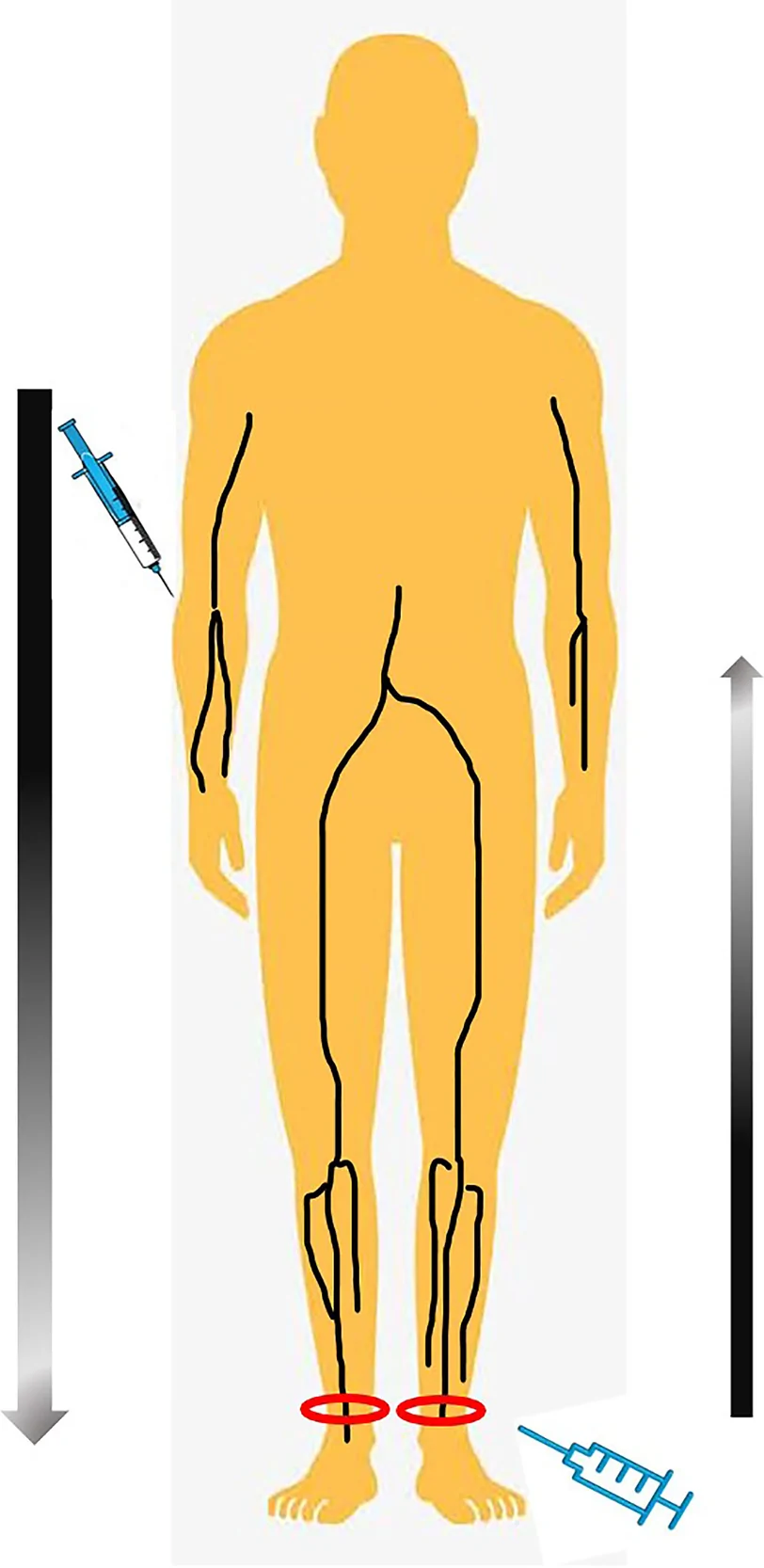
Two methods for lower limb CTV (contrast-enhanced tomography) examination. First is the direct method, where contrast agent is injected from the dorsum of the foot, with gradually decreasing concentration and poor filling from the distal calf to the inferior vena cava. The second method involves injection from the brachiocephalic vein, scanning the pulmonary artery and lower limb veins, with good visualization of the pulmonary artery and gradual concentration decrease from the proximal to the distal calf of the lower limb veins.

However, patients with lower limb DVT are prone to concurrent pulmonary embolism. Given the principles of deep learning algorithms, separate models are typically required to screen for thrombi in different anatomical sites (PE and DVT) by CT or ultrasound imaging, or multi-model imaging (3–8). In clinical PE imaging, diagnosis is often relatively straightforward due to the confined pulmonary vascular territory and higher contrast agent concentration. In contrast, for lower limb CTV, despite guidelines recommending indirect scanning methods, the long path through both lower extremities leads to a significant decrease in contrast agent density, adversely affecting image quality and reducing thrombus detection rates (Figure 1). This study aims to investigate two deep learning approaches: one involves developing a deep learning model directly from lower limb DVT data; the other involves developing a model from PE data and subsequently adapting it for lower limb DVT screening through enhanced learning. The performance of both models will be compared against that of radiologists to evaluate the accuracy of deep learning models in diagnosing DVT.

## Materials and methods

### Study population and data collection

The study received approval from the ethics committee. This retrospective study was approved by our institutional review board, and the requirement for written informed consent was waived. The article was prepared in accordance with the Standards for Reporting Diagnostic Accuracy Studies (STARD).

We retrospectively collected data from 119 patients diagnosed with lower limb DVT who underwent lower limb CTV examination and 40 patients with negative DVT between April 2021 and July 2024. Additionally, 111 patients diagnosed with PE by computed tomographic pulmonary angiography (CTPA) and 20 patients with negative PE during the same period were enrolled. The diagnostic criteria for thrombosis are: filling defects observed in the pulmonary artery or lower limb venous vessels. For lower limb venous thrombosis, additional imaging modalities such as ultrasound and D-dimer testing are also employed.

### Image acquisition protocols

Lower Limb CTV: All patients were scanned using a Philips Incisive CT scanner. Scanning parameters: tube voltage 120 kVp, automatic tube current modulation, slice thickness 0.9 mm, slice increment 0.45 mm, collimation width 64 × 0.625 mm. The direct venography method was employed: 60 mL of non-ionic contrast agent iohexol (350 mg I/mL) was diluted with normal saline at a 5:1 ratio. Using a dual-syringe power injector in dual-flow mode, 180 mL of the diluted contrast agent was administered via a dorsal pedal vein in each lower limb (total injection rate 4.6 mL/s, with a unilateral limit of 2.3 mL/s). Scanning commenced with a 20 s delay after the start of contrast injection, concluding simultaneously with the end of the injection. The scan coverage extended from the ankle joint to the diaphragm. Patients were positioned supine, and tourniquets were applied moderately at both ankle joints prior to injection to prevent superficial venous reflux.

CTPA: All patients were scanned using a second-generation Siemens dual-source CT scanner in the supine position, with coverage from the lung apex to the base. Scanning parameters: slice interval 0.75−1.0 mm, reconstructed slice thickness 0.75−1.0 mm, collimation 128 × 0.6 mm. Contrast-enhanced scanning was performed by injecting 35−40 mL of non-ionic contrast agent iohexol (350 mg I/mL) via an antecubital vein at a rate of 4−5 mL/s. Scanning was performed during a single end-expiratory breath-hold, lasting 4−7 s.

Conventional Imaging Assessment and Thrombus Annotation.

A reference standard was established by a radiologist with over 10 years of diagnostic experience who had access to all patients' clinical information and ultrasound reports. This radiologist meticulously annotated all thrombi by drawing bounding boxes (BBOX) encompassing the thrombus area on every slice. Vascular segments were categorized into three groups: pelvic segment (including venae cava inferior (IVC), common iliac vein (CIV), internal iliac vein (IIV), external iliac vein (EIV), F-P segment [including femoral vein (FV), popliteal vein (PV)], and calf segment [including anterior tibial vein (ATV), posterior tibial vein (PTV), peroneal vein (PNV)]. Additionally, the same radiologist annotated thrombi on CTPA images. All annotations for both lower limb CTV and CTPA were subsequently reviewed and corrected by a second radiologist with over 10 years of experience.

### Study design and procedures

**Figure 2:**
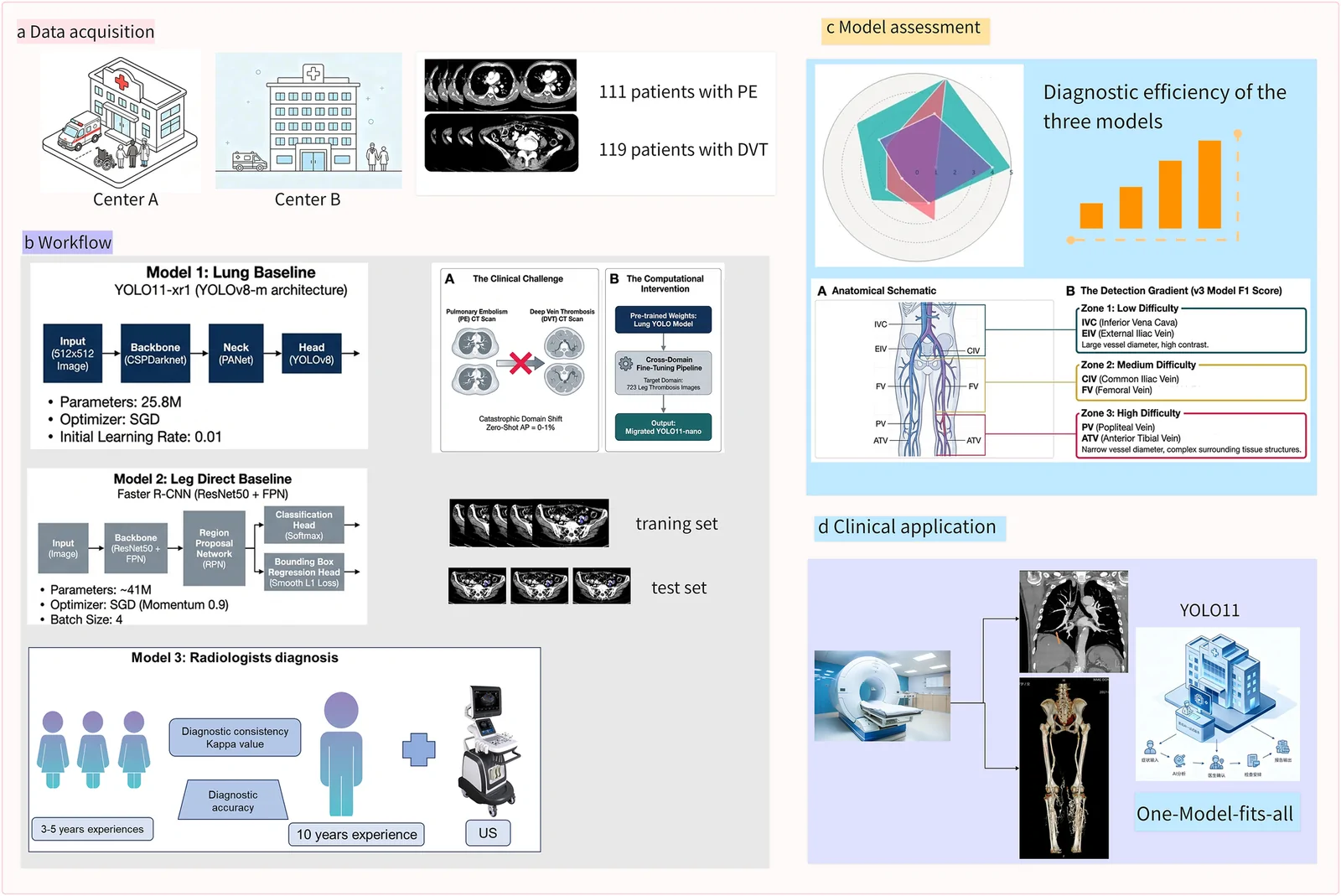
The flow chart of the study. *DVT*, Deep vein thrombosis *PE*, pulmonary embolism.

This study comprised three parts, with the detailed workflow illustrated in Figure 2.

Part I: Assessment by Resident Radiologists.

Three resident radiologists with 3−5 years of diagnostic experience independently evaluated the 119 CTV examinations. They assessed the presence of thrombi in the predefined lower limb venous segments: IVC, CIV, EIV, IIV, FV, PV, ATV, PTV, and PNV. The residents were blinded to clinical information, original radiology reports, and other imaging findings.

#### Evaluation metrics (part I)

Standard object detection metrics were used, including accuracy, sensitivity, specificity, precision, recall, and F1-score, defined as follows:

$$\begin{array}{rl} \mathrm{Precision}= & \frac{\mathrm{TP}}{\mathrm{TP}+\mathrm{FP}}\\ \mathrm{Recall}= & \frac{\mathrm{TP}}{\mathrm{TP}+\mathrm{FN}}\\ {}*F1-\mathrm{Score}\;= & \;\frac{2\times (\mathrm{Precision}\times \mathrm{Recall})}{\mathrm{Precision}+\mathrm{Recall}} \end{array}$$

where TP (True Positive) is the number of correctly detected thrombi, FP (False Positive) is the number of incorrectly detected thrombi, and FN (False Negative) is the number of missed thrombi.

Part II: Deep Learning-Based Single-Target Detection Model for DVT.

The dataset for this part comprised 1,171 labeled images from 119 patients. All images were uniformly resized to 512 × 512 pixels and stored in JPG format.

Dataset Splitting: To ensure objective model evaluation, the dataset was randomly split into training, validation, and test sets in a 70:15:15 ratio. The training set (*n* = 723 images), validation set (*n* = 225 images), and test set (*n* = 223 images). The test set was kept completely unseen during training. Basic preprocessing was applied, but no additional data augmentation strategies were employed to preserve the integrity of medical image features. Each patient was assigned exclusively to the training, validation, or test set, and all CT images from that patient were retained in the same subset. Model fitting used only the training set, model and threshold selection used only the validation set, and the test set was evaluated once after all rules had been locked.

Model Architecture: The Faster R-CNN framework (9) was selected as the base detection architecture. Faster R-CNN is a two-stage detector comprising a Region Proposal Network (RPN) and a Fast R-CNN detection network, known for high precision and accurate localization, making it suitable for medical imaging. ResNet-50 (10) was used as the backbone network, with features extracted and fed into a Feature Pyramid Network (FPN). Candidate regions generated by the RPN were then used for classification and bounding box refinement. Given the relatively small medical dataset, a transfer learning strategy was employed, initializing the model with weights pre-trained on the COCO dataset and then fine-tuning on the thrombus dataset.

#### Evaluation metrics (part II)

The same accuracy, sensitivity, specificity, precision, recall, and F1-score metrics were used. Additionally, Intersection over mean Average Precision at IoU threshold 0.5 (mAP@0.5) and mAP50-95 were introduced. mAP50 and mAP50-95 are core metrics in object detection, assessing both the correctness and localization accuracy of predictions (9).

Part III: Transfer Learning from PE Model to DVT.

First, CTPA dataset from 111 patients diagnosed with PE was randomly split into training, validation, and test sets following a 70:15:15 ratio, ensuring no data leakage between subsets. This model was tested in private data and identified in public data. Second, the lower limb CTV dataset was split as described in Part II.

Model Architecture Analysis: The pulmonary model utilized YOLOv8-m (11, 12) as the base architecture. This model was chosen for its suitability for small-to-medium scale datasets (reducing overfitting risk), fast training speed, high iteration efficiency, and strong baseline performance. YOLO11-nano, migrated model from YOLOv8-m, was adapted to the lower limb dataset through two training stages: rapid learning and fine-tuning. Explore optimal training strategies to achieve the best model performance. Evaluation metrics were identical to those in Part II.

### Statistical analysis

Cohen's *κ* and Fleiss' *κ* were used to evaluate inter-rater diagnostic consistency among the three readers. Agreement values were interpreted as: 0–0.20 slight, 0.21–0.40 fair, 0.41–0.60 moderate, 0.61–0.80 substantial, and 0.81–1.00 almost perfect agreement (13). A two-tailed *P*-value <  0.05 was considered statistically significant. For each thrombus segment, diagnostic precision, sensitivity, and balanced accuracy were calculated based on true positives, false positives, and false negatives per segment.

## Results

Initially, 563 patients with CTV images were collected. A total of 119 patients with positive DVT and 40 patients with negative DVT were ultimately included in the study after excluding 121 patients due to poor image quality, 151 due to insufficient imaging coverage, and 132 due to equivocal thrombus diagnosis. Supplementary Table S1 summarizes the demographic and clinical characteristics of the study patients.

Faster R-CNN model and YOLOv8-m model performed an accuracy of 99.1% & 98.4%, a sensitivity of 97.5% & 96.9%, and a specificity of 100% & 100%, respectively in diagnosing PE. It attained an mAP50 of 0.3146, which was lower than the YOLO tested models (mAP50 was 0.4238). YOLO11-nano model was conducted from 100 to 300 epochs (in four stages) to choose optimal performance, with 200 epochs leading to best performance.

Diagnostic Performance of Radiologists and Deep Learning Algorithms.

The overall performance of the three methods on the test set is presented in Table 1, along with segment-wise performance. Cohen's *κ* coefficient for pairwise agreement among the three resident radiologists ranged from 0.56 to 0.81. For each lower limb segment, Fleiss' *κ* coefficient ranged from 0.45 to 0.55.

**Table 1:** Performances of deep learning algorithms and radiologists.

Performances of deep learning algorithms and radiologists were compared	Segment	Faster R-CNN	YOLO11-nano	Reader1	Reader2	Reader3
Accuracy	Pelvic segment	56%	77.8%	96.2%	96.9%	95.1%
	F-P segment	48%	77.2%	93.5%	91.8%	91.2%
	Calf segment	34%	66.7%	84.0%	87.4%	81.4%
	Total (patient level)	66%	92.1%	89.0%	92.8%	90.3%
	PE	99.1%	98.4% (YOLOv8-m)	-	-	-
Sensitivity	Pelvic segment	69%	71.1%	95.1%	94.3%	86.1%
	F-P segment	70%	72.3%	93.5%	94.3%	86.6%
	Calf segment	57%	75.0%	58.1%	96.4%	58.9%
	Total (patient level)	68%	93.9%	81.9%	95.1%	77%
	PE	97.5%	96.9% (YOLOv8-m)	-	-	-
Specificity	Pelvic segment	60%	88.0%	85.8%	97.9%	99%
	F-P segment	62%	85.1%	93.5%	88.8%	96.7%
	Calf segment	43%	59.1%	98.3%	82.4%	93.7%
	Total (patient level)	60%	80.2%	92.5%	91.7%	97.2%
	PE	100%	100% (YOLOv8-m)	-	-	-
Precision (P)	Pelvic segment	75.2%	90.0%	92.1%	97.2%	95.0%
	F-P segment	70.2%	88.7%	94.6%	97%	91.1%
	Calf segment	61.7%	62.5%	85.5%	83.7%	75.1%
	Total (patient level)	73.5%	87.5%	81.9%	93.3%	86.0%
Recall (R)	Pelvic segment	82.1%	87.2%	95.1%	86.1%	94.2%
	F-P segment	85.6%	86.1%	93.5%	86.6%	94.3%
	Calf segment	54%	57.7%	58.1%	58.9%	96.4%
	Total (patient level)	79.3%	88.6%	73.6%	77.2%	95.0%
mAP@0.5	IoU=0.5	31.46%	42.38%			

PE, pulmonary embolism. Pelvic segment includes venae cava inferior, common iliac vein, internal iliac vein, external iliac vein. Femoral- poplitea*l* segment includes femoral and popliteal vein. Calf segment includes anterior tibial vein, posterior tibial vein, peroneal vein. F-P: Femoral- popliteal. F1-Score = 2 × P × R/(P + R) Total (patient level) As long as any segment is correctly classified, it is considered accurate for that patient.

**Figure 3:**
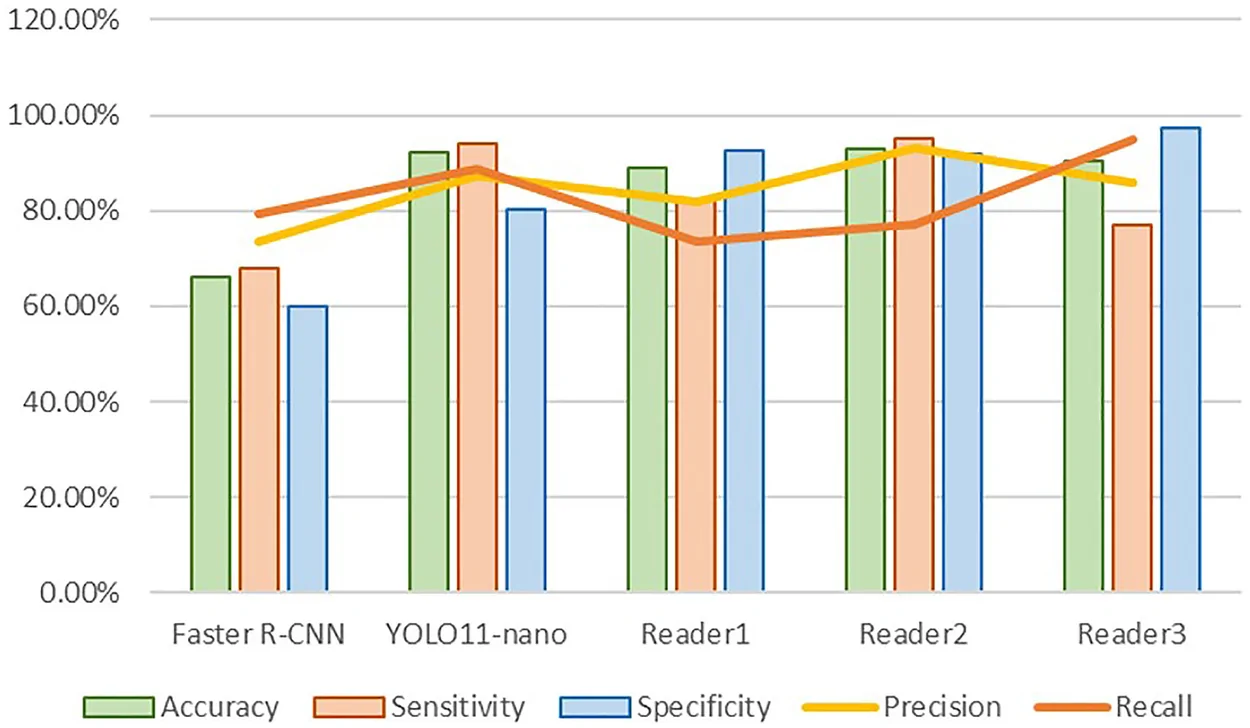
Comparison of the overall diagnostic performance of three types of radiologists and two models in the diagnosis of deep vein thrombosis (DVT) in the lower extremities.

The accuracy and sensitivity of YOLO11-nano were close to the best residential radiologist(92.1% & 92.8%, 93.9% & 95.1%), better than Faster R-CNN models, suggesting they can detect the vast majority of thrombi with low miss rates (Figure 3). The Faster R-CNN model had the lowest specificity among the three methods, indicating the highest rate of false positives.

**Figure 4:**
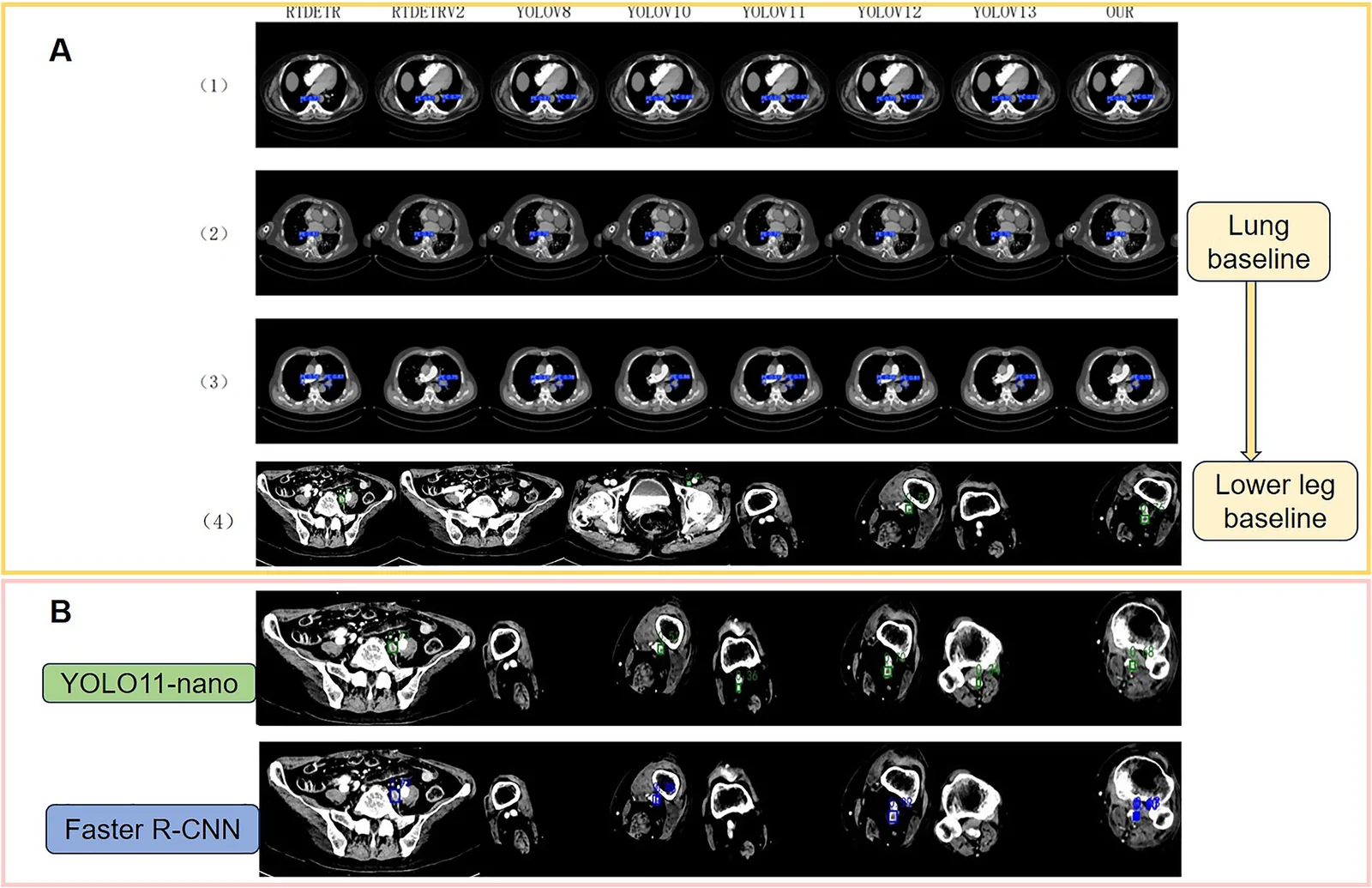
Thrombus labeling status on both models. In panel **(A)**, the comparison between readers and all YOLO models (line 1-3) was to select the best YOLO models for pulmonary embolism, and later adapted via transfer learning for detecting deep vein thrombosis (DVT) in the lower extremities (line 4). In panel **(B),** in DVT detection, green bounding boxes were generated via YOLO11-nano, whereas bule bounding boxes were produced using R-CNN. Additionally, we have adjusted some color in Panel B to suit for our descriptions.

Regarding segment-wise diagnostic performance, all three methods showed different accuracy and sensitivity for thrombi in the pelvic and femoropopliteal segments, with the lowest performance in the calf segment. The Faster R-CNN model consistently underperformed compared to the YOLO11-nano model across all segments. Accuracy, sensitivity, and specificity were lower than all the radiologists (Figure 4).

## Discussion

In this study, the YOLO model demonstrated exceptional performance, capable of diagnosing in PE as Faster R-CNN. Both the YOLO11 nano model and the Faster R-CNN model exhibited high stability in the initial diagnosis of PE. By contrast, YOLO11 nano showed a relatively high accuracy, sensitivity, and specificity in the overall diagnostic capability in lower extremity DVT compared to radiologists, yet remains superior to the Faster R-CNN. Although YOLO's diagnostic performance is inferior to that of radiologists at each segment, it reaches 77% accuracy in the pelvic and femoral-popliteal segments, demonstrating the feasibility of the transfer learning model for simultaneous diagnosis of PE and DVT.

In the Chinese guidelines for the diagnosis of lower extremity venous thrombosis, it is recommended that the combined use of CTV and CT pulmonary angiography can improve the diagnostic rate of VTE (14). Research on the application of deep learning models in lower extremity deep vein thrombosis has gradually increased, and the findings suggest that deep learning models can enhance the detection rate of thrombosis (6, 15, 16).

In this study, we selected the Faster R-CNN and YOLO models for lower extremity thrombus target detection, both based on algorithms from previous literature (6, 8, 17). In direct lower extremity CTV imaging, a high-pressure injector is used to inject contrast agent via the dorsal vein of the affected foot, allowing antegrade visualization of the contrast agent and displaying only the lower extremity veins. Both models were able to directly detect PE with high diagnostic sensitivity (Faster R-CNN: 97.5%, YOLO11-nano: 96.9%). These results are consistent with the sensitivity range of 77%–98% reported in the literature, demonstrating significant efficiency and stability for thrombus detection (15, 18–21) (Supplementary Table S2). For thrombus detection in different segments of the lower extremity veins, both precision and accuracy exceeded 75% for the pelvic and femoropopliteal segments, indicating good diagnostic performance, although lower than residential radiologists. Much more data would be used to training the model for improving the accuracy. It is worth noting that the detection accuracy rate for calf vein thrombi was relatively low among junior physicians, at approximately 80%, primarily due to the small and complex nature of these vessels and the relatively low contrast agent density in this region. However, the application of the deep learning model significantly improved the thrombus detection rate in this segment, reaching 67%.

The application of machine learning in clinical practice is becoming widespread, with numerous software tools and imaging plug-ins emerging. Often, a specific tool is required for each disease. The ability to integrate or make these tools compatible and generalizable across different anatomical sites for similar disease processes (e.g., VTE) could become a future necessity to artificial intelligence. A key challenge is achieving cross-domain generalization without compromising performance. Although PE and lower limb DVT are manifestations of the same disease (VTE), appearing as filling defects within opacified vessels, the underlying image characteristics differ significantly for deep learning models. Pulmonary arteries are horizontally oriented within air-filled lung tissue, whereas lower limb veins are vertically oriented within muscle and fat. Additionally, contrast concentrations and scan phases differ. Overcoming this domain shift is a significant challenge. The YOLO11-nano model (10, 12) demonstrated the ability to address these issues, showing compatibility with datasets from different anatomical sites, enabling rapid training, efficient iteration, and suitability for small-to-medium datasets with reduced overfitting risk. Through transfer learning with YOLO11-nano, this study successfully created a model capable of detecting both PE and DVT, corroborating the feasibility of this approach (22). Importantly, the PE and DVT datasets originated from two different study centers, helping to mitigate potential model bias and overfitting.

## Limitations

This study has several limitations. First, the sample size for lower limb DVT remains modest; larger datasets are needed to further enhance model performance and generalizability. As suggested by Reyes Gil et al. (23) and Altememi et al. (24, 25), external validation and prospective testing of AI/ML models are crucial to reduce bias risk. Although utilized datasets from two centers, external validation was not performed, leaving a risk of overfitting. Future work should include prospective validation cohorts. Second, the pulmonary and lower limb datasets were used separately; we did not experiment with merging data from both sites to train a unified, general thrombus detection model. Such an approach could potentially improve performance further. Future efforts could also incorporate thrombus datasets from other anatomical locations, which may be significant for enhancing detection accuracy across the VTE spectrum.

## Conclusion

This study developed a YOLO11-nano model based on transfer learning from a pulmonary embolism dataset. The YOLO11-nano model demonstrated advantages including high sensitivity and high diagnostic consistency, significantly improving the detection rate for deep vein thrombi in the calf segment. This model could potentially assist in the preliminary screening of patients suspected of having concomitant lower limb DVT and PE, playing a key role in enabling radiologists to rapidly diagnose and localize VTE.

## Data Availability

The raw data supporting the conclusions of this article will be made available by the authors, without undue reservation.

## References

[1] Nyamekye IK. European society for vascular surgery (ESVS) 2022 clinical practice guidelines on the management of chronic venous disease of the lower limbs. JMV. (2022) 47(2):53–5. 10.1016/j.jdmv.2022.04.003 35691663

[2] Kolh P Verhagen HJM. The ESVS guidelines provide a solid scientific basis to treat patients with an abdominal aorto-iliac aneurysm in Europe and beyond. Eur J Vasc Endovasc Surg. (2021) 62(6):845–6. 10.1016/j.ejvs.2021.09.012 34736845

[3] Du H Sui X Zhao R Wang J Ming Y Piao S. A comparative analysis of deep learning and hybrid iterative reconstruction algorithms with contrast-enhancement-boost post-processing on the image quality of indirect computed tomography venography of the lower extremities. BMC Med Imaging. (2024) 24(1):163. 10.1186/s12880-024-01342-0 38956583 PMC11218076

[4] Joseph BR Jebadurai IJ Paulraj GJ Jebadurai J Varuvel MM. Deep vein net: deep vein throbosis identification via sooty tren optimized deep learning network. Rev Roum Sci Techn– Électrotechn et Énerg. (2024) 69(1):115–20. 10.59277/RRST-EF.2024.1.20

[5] Tharek AB Noralam NHA-A Perisamy RS Ismail LI Hui S Muda AS. Automated detection of hemorrhage and infraction on diffusion-weighted MRI: comparative performance of YOLOv8, FASTER R-CNN, and radiologists. Xi'an Shiyou Daxue Xuebao (Ziran Kexue Ban). (2025) 68:36–52. 10.5281/zenodo.17347379

[6] Hwang J Seo J Kim J Park S Kim Y Kim K. Comparison between deep learning and conventional machine learning in classifying iliofemoral deep venous thrombosis upon CT venography. Diagnostics. (2022) 12(2):274. 10.3390/diagnostics12020274 35204365 PMC8871174

[7] Tian Y Liu J Wu S Zheng Y Han R Bao Q. Development and validation of a deep learning-enhanced prediction model for the likelihood of pulmonary embolism. Front Med (Lausanne). (2025) 12:1506363. 10.3389/fmed.2025.1506363 39981086 PMC11839595

[8] Chen P-W Tseng B-Y Yang Z-H Yu C-H Lin K-T Chen J-N. Deep learning model for diagnosis of venous thrombosis from lower extremity peripheral ultrasound imaging. iScience. (2024) 27(12):111318. 10.1016/j.isci.2024.111318 39687013 PMC11647138

[9] Ren S He K Girshick R Sun J. Faster R-CNN: towards real-time object detection with region proposal networks. IEEE T Pattern Anal. (2017) 39(6):1137–49. 10.1109/TPAMI.2016.2577031 27295650

[10] Liang L Wang C Zhang L. MambaFPN: a SSM-based feature pyramid network for object detection. Neural Netw. (2026) 198:108544. 10.1016/j.neunet.2026.108544 41579822

[11] Jiang Y Chen X Ge H Wen X Jiang M Zhang Y. THz image recognition of moldy wheat based on multi-scale context and feature pyramid. Front Plant Sci. (2025) 16:1490384. 10.3389/fpls.2025.1490384 40535927 PMC12174122

[12] Shen A Li C Yang J Huang G Zhang X. YOLO-VML: an improved object detection model for blastomeres and pronuclei localization in IoMT. IEEE Open J Eng Med Biol. (2026) 7:35–42. 10.1109/OJEMB.2025.3644699 41669278 PMC12885487

[13] Landis JR Koch GG. The measurement of observer agreement for categorical data. Int Biometric Soc. (1977) 33(1):159–74. 10.2307/2529310 843571

[14] Vascular Surgery Group CSoS, Chinese Medical Association. Guidelines for the diagnosis and treatment of deep vein thrombosis (3rd edition). Chin J Gen Surg. (2017) 32(09):807–12. 10.3969/j.issn.1674-7429.2017.04.003

[15] Yang X Yu P Zhang H Zhang R Liu Y Li H. Deep learning algorithm enables cerebral venous thrombosis detection with routine brain magnetic resonance imaging. Stroke. (2023) 54(5):1357–66. 10.1161/STROKEAHA.122.041520 36912139

[16] Butova X Shayakhmetov S Fedin M Zolotukhin I Gianesini S. Artificial intelligence evidence-based current Status and potential for lower limb vascular management. J Pers Med. (2021) 11(12):1280. 10.3390/jpm11121280 34945749 PMC8705683

[17] Anghele A-D Marina V Dragomir L Moscu CA Anghele M Anghel C. Predicting deep venous thrombosis using artificial intelligence: a clinical data approach. Bioengineering. (2024) 11(11):1067. 10.3390/bioengineering11111067 39593727 PMC11590985

[18] Sun C Xiong X Zhang T Guan X Mao H Yang J. Deep learning for accurate segmentation of venous thrombus from black-blood magnetic resonance images: a multicenter study. BioMed Res Int. (2021) 2021:1–11. 10.1155/2021/4989297 34950733 PMC8692022

[19] Yang K-C Xu Y Lin Q Tang L-L Zhong J- An H-N. Explainable deep learning algorithm for identifying cerebral venous sinus thrombosis-related hemorrhage (CVST-ICH) from spontaneous intracerebral hemorrhage using computed tomography. eClinicalMed. (2025) 81:103128. 10.1016/j.eclinm.2025.103128 PMC11909457 40093990

[20] Sun Z Hao D Yang M Wu W Jing H Yang Z. Development and validation of a machine learning model for predicting venous thromboembolism complications following colorectal cancer surgery. Visual Computing for Industry, Biomedicine, and Art. (2025) 8(1):22. 10.1186/s42492-025-00204-y 40936051 PMC12425853

[21] Zhou Y Kuang Y Zhang J Yang C Guo W Liu X. Machine learning-based model for predicting recanalization in isolated distal deep vein thrombosis and analysis of predictors. PLoS One. (2026) 21(5):e0349110. 10.1371/journal.pone.0349110 42102041 PMC13155594

[22] Lee P Bubeck S Petro J. Benefits, limits, and risks of GPT-4 as an AI chatbot for medicine. N Engl J Med. (2023) 388(13):1233–9. 10.1056/NEJMsr2214184 36988602

[23] Reyes Gil M Pantanowitz J Rashidi HH. Venous thromboembolism in the era of machine learning and artificial intelligence in medicine. Thromb Res. (2024) 242:109121. 10.1016/j.thromres.2024.109121 39213896

[24] Altememi MA Favaloro EJ Islam MZ Santhakumar AB. Artificial intelligence and machine learning in thrombosis and hemostasis: a scoping review of clinical and laboratory applications, challenges, and future directions. Clin Chem Lab Med. (2026) 64(4):767–80. 10.1515/cclm-2025-1450 41400592

[25] Lam BD Chrysafi P Chiasakul T Khosla H Karagkouni D McNichol M. Machine learning natural language processing for identifying venous thromboembolism: systematic review and meta-analysis. Blood Advances. (2024) 8(12):2991–3000. 10.1182/bloodadvances.2023012200 38522096 PMC11215191

