# Supplementary material for: Deep learning algorithm enables lower limb venous thrombosis detection with CT venography
Source: Front Cardiovasc Med. 2026 Aug 12;13:1890645. doi: 10.3389/fcvm.2026.1890645 (PMC13507883; doi:10.3389/fcvm.2026.1890645)
Supplement: Supplementary file 1 [file Table1.docx]

**Supplementary Table1 The clinical features of patients with VTE**

|  | 119 cases |
| --- | --- |
| Age, mean (±SD), y | 59.62±12.98 |
| Sex, % (n) |  |
| Female | 46 (38.7%) |
| Male | 73 (61.3%) |
| BMI, mean (±SD) | 27.6±12.3 |
| **DVT extent, % (n)** |  |
| Pulmonary artery | 11 (9.2%) |
| Venae cava inferior | 34 (28.6%) |
| Common iliac vein | 54 (45.4%) |
| Internal iliac vein | 44 (37%) |
| External iliac vein | 74 (62.2%) |
| Femoral vein | 106 (89.1%) |
| Popliteal vein | 106 (89.1%) |
| Peroneal veins | 81 (68.1%) |
| Posterior tibial veins | 88 (73.9%) |
| Anterior tibial veins | 33 (27.7%) |
| **Affected side, % (n)** |  |
| Left | 52 (43.7%) |
| Right | 31 (26.1%) |
| Bilateral | 26 (21.8%) |
| **History** |  |
| Hypertension% | 60 (50.4%) |
| Diabetes | 15 (12.6%) |
| Hyperlipidemia | 4 (3.3%) |
| Tumor patients | 56 (47.1%) |
| Trauma | 13 |
| D-dimer | Two patients<0.55 |
| **Clinical syndrome** |  |
| Chest tightness, shortness of breath | 11 |
| Swelling and pain in the lower limbs | 92 |
| **Treatment** |  |
| Conservative care | 29 |
| Stent placement | 8 |
| Venous thrombectomy | 3 |
| Others | 79 |

*VTE:* *venous thromboembolism BMI: body mass index DVT:* deep vein thrombosis
