# Supplementary material for: Deep learning algorithm enables lower limb venous thrombosis detection with CT venography
Source: Front Cardiovasc Med. 2026 Aug 12;13:1890645. doi: 10.3389/fcvm.2026.1890645 (PMC13507883; doi:10.3389/fcvm.2026.1890645)
Supplement: Supplementary file 2 [file Table2.docx]

**Supplementary Table2** **Previous literatures about model algorithm for deep vein thrombosis using imaging examinations**

| Author (year) | Model | Accuracy | Sensitivity | Specificity | PPV | NPV | F1 Score | Recall | Precision |
| --- | --- | --- | --- | --- | --- | --- | --- | --- | --- |
| Sun C et al (2021) | Based on U-Net | 0.94 | 0.93 | 0.92 | 0.82 | 0.96 |  |  |  |
|  |  |  |  |  |  |  |  |  |  |
|  |  |  |  |  |  |  |  |  |  |
| Sun et al (2025) | LG, RF, SVM, MLP, XGB, CatBoost, Stacking | 0.85-0.97 | 0.54-0.96 | 0.94-0.98 |  |  |  | 0.88-0.98 |  |
| Zhou et al（2026） | LR, SVM, RF, MLP, XGBoost, Voting Ensemble | 0.84-0.90 | 0.54-0.96 | 0.84-0.96 |  |  | 0.51-0.72 | 0.43-0.82 | 0.50-0.67 |

MSMT-DL: multisequence multitask deep learning algorithm

Their model: A transfer learning-based 3D U-Net with segmentation and classification

PPV: positive prediction value NPV: negative prediction value
